# Cognitive Function Deficits Associated With Type 2 Diabetes and Retinopathy: Volumetric Brain MR Imaging Study

**DOI:** 10.1002/brb3.70387

**Published:** 2025-02-28

**Authors:** Ece Ozdemir Oktem, Dila Sayman, Sevilay Ayyildiz, Çaglar Oktem, Lutfiye Ipek, Behcet Ayyildiz, Fatih Aslan, Emin Utku Altindal, Nilay Yagci, Rumeysa Dikici, Ramazan Karaca, Şeyda Cankaya, Seda Avnioglu, Halil Aziz Velioglu, Burak Yulug

**Affiliations:** ^1^ Department of Neurology and Neuroscience Alanya Alaaddin Keykubat University Antalya Turkey; ^2^ School of Medicine, TUM‐NIC Neuroimaging Center Technical University of Munich Munich Germany; ^3^ Department of Ophthalmology Alanya Alaaddin Keykubat University Antalya Turkey; ^4^ Department of Anatomy Kocaeli University Kocaeli Turkey; ^5^ Department of anatomy Alanya Alaaddin Keykubat University Antalya Turkey; ^6^ Center for Psychiatric Neuroscience Feinstein Institute for Medical Research Manhasset New York USA

**Keywords:** MRI, retinopathy, type 2 diabetes

## Abstract

**Introduction:**

Type 2 diabetes mellitus is a ubiquitous chronic inflammatory disease with deleterious effects on various tissues, including the kidney, retina, and peripheral nerves. Studies using histopathology and magnetic resonance imaging have revealed that diabetes‐related chronic hyperglycemia may impact the brain's essential functioning by causing microvascular damage. The aim of this study was to examine the cognitive functioning of type 2 diabetic individuals with and without retinopathy by evaluating their morphological, structural, and biochemical differences.

**Methods:**

Demographic characteristics, education level, type of diabetes mellitus (DM), disease duration, treatment received, other diabetic complications, such as nephropathy and neuropathy, and detailed medical histories were recorded. All participants underwent an extensive neuropsychological examination with Montreal Cognitive Assessment (MoCA) testing. Brain magnetic resonance imaging was performed to evaluate gray matter volume differences between the groups.

**Results:**

Gray matter volume differences between the groups were observed. Differences were observed after multiple corrections (age, education, and total intracranial volume [TIV]). First, the diabetic retinopathy group exhibited a significantly smaller gray matter volume in the right inferior temporal gyrus than the diabetic group (*p* = 0.032). In addition, the diabetic retinopathy group exhibited a significantly smaller gray matter volume than the control group in the right insula (lateral and central part) (*p* = 0.011). In addition, MoCA scores exhibited significant correlation with the two regions emerging as statistically significant in our analyses (the right inferior temporal gyrus and right insula) (*p* = 0.003, *p* = 0.002, respectively).

**Conclusion:**

Our results suggest the presence of a neurodegenerative process associated with cognitive dysfunction that is particularly prominent in the retinopathy stage of DM.

## Graphical abstract

This study aimed to assess cognitive functioning in type 2 diabetic individuals with and without retinopathy by evaluating differences in brain morphology, structure, and biochemistry. Participants underwent neuropsychological testing and brain MRI, revealing gray matter volume differences between the groups. The diabetic retinopathy group showed significantly smaller gray matter volume in the right inferior temporal gyrus and right insula compared to both the diabetic group without retinopathy and the control group. These changes were linked to lower cognitive scores, suggesting that neurodegeneration and cognitive dysfunction are more pronounced in individuals with diabetic retinopathy.

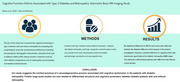

## 1 Research in Context

The aim of this study was to examine the cognitive functioning of type 2 diabetic individuals with and without retinopathy by evaluating their morphological, structural, and biochemical differences evaluating the participants’ demographic characteristics, education level, type of diabetes mellitus (DM), disease duration, treatment received, other diabetic complications such as nephropathy and neuropathy, and detailed medical histories. All participants underwent an extensive neuropsychological examination with Montreal Cognitive Assessment (MoCA) testing. Brain MRI examinations were performed to evaluate gray matter volume differences between the groups and showed gray matter volume differences between the groups. First, the diabetic retinopathy (DMR) group exhibited a significantly smaller gray matter volume in the right inferior temporal gyrus than the DM group (*p* = 0.032). In addition, the DMR group exhibited a significantly smaller gray matter volume than the control group in the right insula (lateral and central part) (*p* = 0.011). In addition, MoCA scores exhibited significant correlation with the two regions emerging as statistically significant in our analyses (the right inferior temporal gyrus and right insula (*p* = 0.003, *p* = 0.002, respectively). Our results suggest the presence of a neurodegenerative process associated with cognitive dysfunction that is particularly prominent in the complicated stage of DM.

## 2 Introduction

Type 2 diabetes mellitus (T2DM) is a ubiquitous chronic inflammatory disease with deleterious effects on various tissues, including the kidney, retina, and peripheral nerves. Studies using histopathology and magnetic resonance imaging (MRI) have revealed that diabetes‐related chronic hyperglycemia may impact the brain's essential functioning by causing microvascular damage (Stumvoll et al. 2005). Diabetic retinopathy (DMR) is one of the most severe neurovascular outcomes of diabetes, causing structural and functional alterations in the retina. In the light of the morphology of retinal ganglion cells synapsing with central structures (such as the lateral geniculate nucleus, mesencephalon, pretectum, and hypothalamus) and the substantial architectural and embryological similarities between retinal and cerebral microvessels, it may be concluded that the retina represents a sizeable proportion of the brain that may indicate subtle changes in cognition (Miller et al. 2010; Patton et al. 2005). One large longitudinal cohort study, for instance, reported a 42% greater risk of onset dementia in patients with DMR (Hugenschmidt et al. 2014). These findings are consistent with recent data indicating a substantial association between DMR and impairment in cognitive functioning in patients with T2DM (Exalto et al. 2014; Stewart and Liolitsa 1999), especially affecting global cognitive function and processing speed. Even in non‐complicated diabetes, numerous neuropsychological investigations have identified mild to moderate cognitive impairments, mainly affecting verbal memory, psychomotor speed, and executive skills (Stewart and Liolitsa 1999; Moran et al. 2017). These conclusions are supported by several epidemiological studies showing a clear relationship between the prevalence of diabetes and dementia (Manschot et al. 2006). Neuroimaging research has repeatedly confirmed that diabetes is associated with critical changes in brain structure and function implicated in cognition, ranging from a total decrease in gray matter volume to a considerable loss in key cognitive brain regions such as the hippocampus, frontal, cingulate, and temporal regions (Moran et al. 2017; Manschot et al. 2006; Lei et al. 2021). Interestingly, most of these studies have indicated accelerated aging of brain regions that are especially prone to DM‐related risk factors, such as hypertension and retinopathy (Moran et al. 2017). One good example of this is reduced white matter volume attributable to microvascular injury observed in the frontal and temporal regions of the brain in patients with DM (Exalto et al. 2014). However, the underlying pathophysiological mechanisms involved in these structural correlations in patients with DM with cognitive impairment are still unknown.

To the best of our knowledge, only a few dynamic imaging studies have revealed significant cognitive network changes in patients with DM before structural changes occur (Lei et al. 2021; Moheet et al. 2015). Based on these findings, the purpose of this research was to examine the cognitive functioning of type 2 diabetic individuals with and without retinopathy by evaluating the morphological, structural, and biochemical differences between them.

## 3 Material and Method

This study was approved by the medical ethics committee of Alanya Alaaddin Keykubat University, Türkiye (Ethical Approval Number: 8–12 3/09/2019). All clinical research procedures were conducted in conformity with the principles of the Declaration of Helsinki, and informed written consent was obtained from all participants. The participants’ demographic characteristics, education levels, types of DM, disease duration, treatments received, other diabetic complications such as nephropathy and neuropathy and detailed medical histories were recorded.

### 3.1 Patient Selection

Twelve patients with T2DM without DMR, 11 patients with DMR, and 15 age‐ and gender‐matched healthy controls were included in the study.

Participants younger than 75 and older than 20 were enrolled. Our exclusion criteria were as follows:

- patients with type 1 diabetes,
- individuals with a severe psychiatric or neurological disorder capable of affecting cognitive functions (such as Alzheimer disease or schizophrenia),
- alcohol or substance abuse,
- mental disability,
- the presence of severe loss of vision or hearing capable of affecting neurocognitive test performance,
- history of intracranial tumor,
- history of a major surgery,
- history of multiple sclerosis, epilepsy, stroke, hemorrhage, any metabolic disease, or any structural lesions that might affect the volumetric measurements.

### 3.2 Ophthalmological Examination

All participants underwent detailed ophthalmologic examinations, including best‐corrected visual acuity measurement using the Snellen scale; anterior segment examination performed with a biomicroscope, intraocular pressure measurement involving a pneumatic tonometer, and dilated fundus examination. Syclopentolate hydrochloride 1% and 1% tropicamide drops were used with a 10‐min interval. Diabetic retinopathy was determined from retinal eye photographs (macula and disc centered) by three ophthalmologists based on the Early Treatment of Diabetic Retinopathy Study grading criteria. A TOPCON TRC 50 DX (Topcon Corporation, Tokyo, Japan) high‐resolution digital retinal camera (3,872 3 2,592 pixels) was used for fundus assessment. Retinopathy status was determined based on the severity of the worse affected eye. Participants also underwent ophthalmic examinations, including slit lamp biomicroscope and ophthalmoscopy.

The patients with DMR were categorized into non‐proliferative DMR and proliferative retinopathy groups according to the Early Treatment Diabetic Retinopathy Study International Classification of Diabetic Retinopathy (Early Treatment Diabetic Retinopathy Study Research Group 1991). Initially, we classified our diabetic retinopathy group into non‐proliferative (*n* = 9) and proliferative (*n* = 2) categories. However, due to the small sample sizes, statistical analysis was not feasible, so we decided to combine them into a single group and assess them as diabetic retinopathy overall.

### 3.3 Cognitive Evaluation

All participants underwent an extensive neuropsychological examination tapping the major cognitive domains in both a verbal and non‐verbal manner. All the tasks were administered in a fixed sequence that took 50 min to complete. The test battery for cognitive function included the Mini‐Mental State Exam (MMSE) and the MoCA test (Nasreddine et al. 2005).

### 3.4 Neuroimaging and Volumetric Measurement

The brain MRI examination was performed using a 1.5‐T MRI device (GE, SIGNA Explorer, General Electric, Milwaukee, USA). Conventional brain MRI on a steady‐state (3D T1 FSPGR) array plus T1‐weighted 3D fast decaying gradient recall procedure was applied for use in segmental volume calculation. The parameter of the 3D T1 FSPGR array was TE 1.7 ms, TR 5.95 ms, flip angle 12°, acquisition matrix 256 × 256, Field of view (FOV) 256 mm^2^, number of slices 170, and slice thickness 1.0 mm

### 3.5 Data Processing

Cortical thickness, surface area, and gray matter volume analyses were performed based on the 3D‐T1–weighted anatomical images using the FreeSurfer software package 7.4.0 (http://surfer.nmr. mgh.harvard.edu/). The recon‐all pipeline in the FreeSurfer was used to segment all participants’ whole brains. Briefly, the automated recon‐all pipeline includes the following steps for segmentation: motion correction, averaging of multiple volumetric T1‐weighted images, skull striping using a deformable template model, automated registration to the Talairach space, segmentation of the subcortical white matter and deep gray matter volumetric structures, normalization of intensity, tessellation of the gray and white matter boundaries, automated topology correction, and surface deformation following intensity gradients for the optimal determination of the gray/white and gray/cerebrospinal fluid borders (Zhang et al. 2020). The entire segmentation procedure was inspected visually and manual corrections were performed as necessary by adding control points (Fischl et al. 2002). Following the registration, the surface was sub‐divided into 148 regions (74 per hemisphere) in the parcellation scheme using the Destrieux Atlas. In the current study, we computed the mean cortical volume (in mm^3^), surface area (in mm^2^), and cortical thickness (in mm) extracted for each of the regions using the cortical parcellation based on that atlas. An intracranial volume (ICV) value was also obtained for each participant. The entire segmentation procedure was visually inspected and manual corrections were made by adding control points when necessary (Fischl et al. 2002). Following registration, the surface was divided into 148 regions (74 per hemisphere) on a subdivision scheme using the Destrieux Atlas. In this study, the average extracted cortical volume (in mm^3^), surface area (in mm^2^), and cortical thickness (in mm) were calculated for each region using cortical parcellation based on this atlas. ICV value was also obtained for each participant.

### 3.6 Blood Biochemistry Assessment

Routine biochemical, complete blood count (CBC), and endocrinological tests, including alanine aminotransferase (ALT), aspartate aminotransferase (AST), creatinine, serum sodium and potassium levels, fasting glucose, HbA1c levels, fasting triglycerides, and fasting cholesterol levels, were performed after 10‐h fasting.

### 3.7 Statistical Analysis

Variables were expressed quantitatively using centralization and measures of variance—mean ± standard deviation (SD). Fisher's exact and Chi‐square tests were used to identify differences in ratios or relationships between categorical variables. ANCOVA analysis was applied to reduce the possible effects of covariance (age, education, and estimated total intracranial volume [eTIV]) of continuous variables and to compare covariance effects across the groups. Assumptions such as homogeneity of the groups, normal distribution of errors, and equality of regression slopes were all included. Multiple comparisons between the groups were corrected via the Bonferroni post hoc correction method. Descriptive values were expressed as mean ± SD and categorical variables as frequency (*n*) and percentage (%). The non‐parametric Spearman's Rank Correlation test was applied to identify correlations between two numerical variables. Vertex‐wise analyses were conducted on FreeSurfer to compare gray matter volume differences among the groups. A general linear module was used to compare cortical thickness, surface area, and gray matter volume among the control, DM, and DMR groups by controlling age, education, and eTIV as covariates. Descriptive statistics, intergroup analyses, and linear relationships between MoCA scores and regional volumes were determined using Jamovi (version 2.3.19.0) (The Jamovi Project [2022]). Jamovi (version 2.3, n.d.). *p* values less than 0.05 were interpreted as statistically significant.

## 4 Results

**FIGURE 1:**
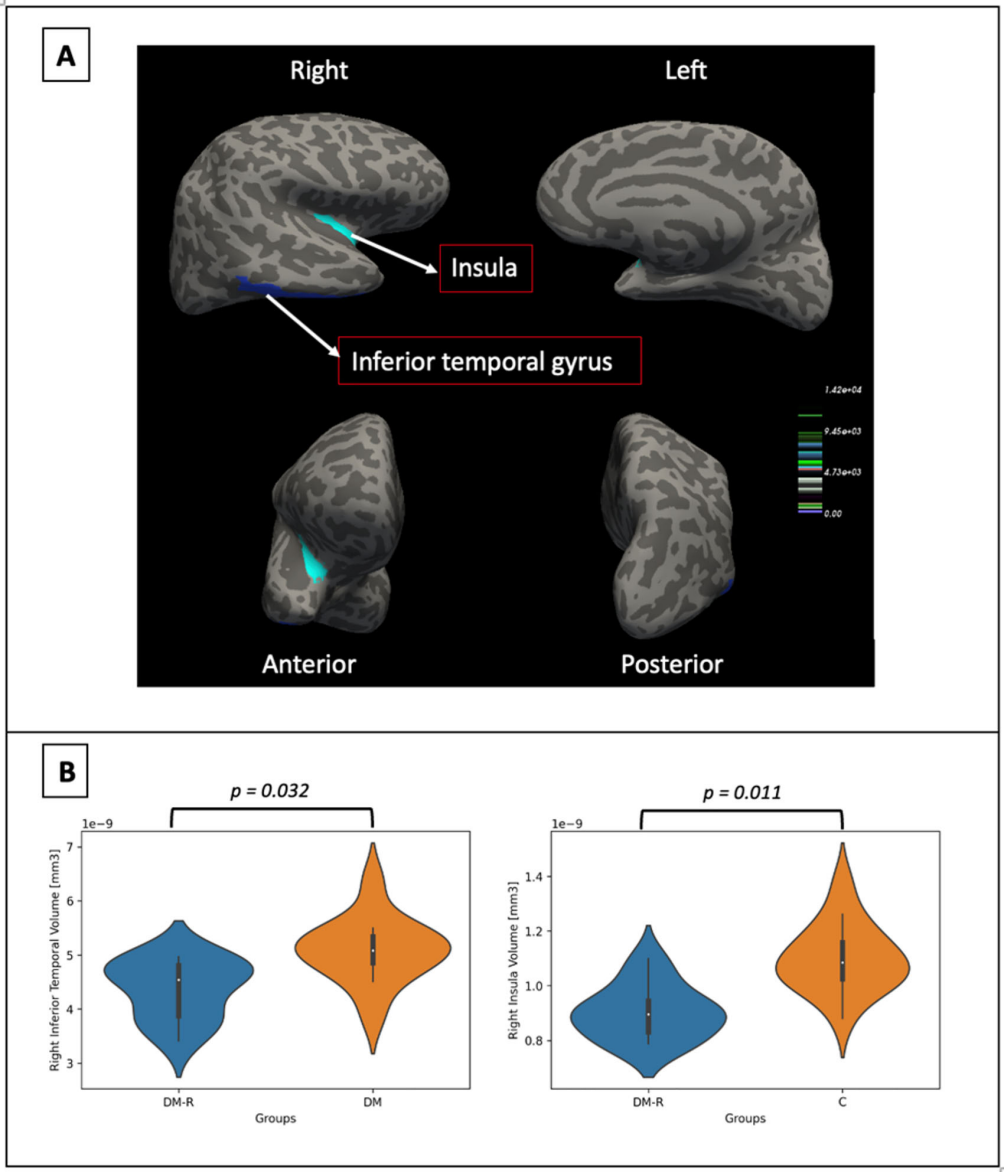
Group comparison of gray matter volume analyses. (A) Brain regions with cortical volume differences between the DMR, DM, and control groups (corrected with Monte–Carlo null‐Z simulation of p < 0.05). Blue (negative values) represents decreased cortical volume in diabetic retinopathy patients. (B) Left panel: Violin plots of bilateral right inferior temporal gyrus volumes derived from manual segmentations and divided by total intracranial volume (TIV) for individuals with DMR and DM individuals TIV (DMR<DM: *p* = 0.032). Right panel: Violin plots of bilateral right insula volumes derived from manual segmentations and divided by TIV are shown for patients with DMR and the control individuals (DMR< C: *p* = 0.011). C, control group; DM, diabetes mellitus patients; DMR, diabetic retinopathy patients.

This study evaluated the data from 15 control subjects and two study groups of patients with DM and DMR, consisting of 12 and 11 individuals, respectively. Descriptive data from the DM, DMR, and control groups in terms of age, sex, other complications such as nephropathy and neuropathy, and treatment options are summarized in Table 1. The mean age of the participants was 51.7 ± 14.5 years, and mean duration of disease was 15.0 ± 8.4 years. Mean cognitive test, age, HbA1c level, and duration of disease values in the study groups are also shown in Table 1. HbA1c levels and MoCA scores differed significantly different between the groups (*p* < 0.001). MoCA scores were significantly lower in the DMR group than in the controls (Table 1). Significant differences in terms of mean age were also observed between the control group and the DM and DMR groups, which led us to evaluate age‐adjusted values exhibiting significant differences in critical brain regions, as described below. Whole brain vertex‐wise analysis revealed gray matter volume differences among the groups. Differences were recorded after multiple corrections (age, education, and total intracranial volume [TIV]). First, the DMR group exhibited a significantly smaller gray matter volume in the right inferior temporal gyrus than the DM group (*p* = 0.032). Moreover, the DMR group exhibited a significantly smaller gray matter volume in the right insula (lateral and central part) than the control group (*p* = 0.011) (all clusters were corrected with Monte Carlo simulations of *p* < 0.05) (Table 2, Figure 1).

**TABLE 1:** Participant characteristics, cognitive test scores, and HbA1c levels in the DM and DMR groups.

		*n* (%)		*p* ^a^	*p* ^b^	*p* ^c^
Participants	DM	12 (32%)				
	DMR	11 (29%)				
	C	15 (39%)				
Gender	Female	20 (53%)				
	Male	18 (47%)				
	**Group**	**Mean ± SD**	**Min–Max**			
Duration (year)	DM	11.3 ± 8.6	1**–**30			
	DMR	18.9 ± 6.3	5**–**30			
Age	DM	55.7 ± 12.8	20**–**68	0.052		
	DMR	58.7 ± 5.9	51**–**68			
	C	43.2 ± 16.4	28**–**75			
Education	DM	8.8 ± 4.4	5**–**16	**0.041**		
	DMR	7.5 ± 2.0	5**–**12	**0.045** ^3^		
	C	12.2 ± 4.6	5**–**16			
MoCA	DM	22.2 ± 4.3	12**–**28	**0.001**	**0.001^x^**	**0.006^1^**
	DMR	16.9 ± 3.2	13**–**22	**0.022** ^1^	0.116^y^	0.660^2^
	C	25.2 ± 3.0	19**–**30	**0.001** ^3^	0.066^z^	**0.001^3^**
HbA1c	DM	6.8 ± 1.0	5.46**–**9.20	**0.001**		
	DMR	8.7 ± 1.7	6.62**–**11.49	0.004^2^		
	C	5.5 ± 0.6	4.39**–**6‐65	0.001^3^		
MMSE	DM	25.5 ± 3.8	17**–**29	**0.009**	0.230^x^	0.513^1^
	DMR	23.7 ± 3.0	18**–**28	**0.004** ^3^	**0.044^y^**	0.726^2^
	C	27.6 ± 1.7	23**–**30		0.418^z^	0.204^3^
R Temporal İnf	DM			**0.027**	**0.029^x^**	**0.032^1^**
	DMR			**0.024** ^3^	0.980^y^	0.978^2^
	C				**0.007^z^**	0.072^3^
R Insula	DM			**0.011**	**0.014^x^**	0.095^1^
	DMR			**0.010** ^3^	0.881^y^	0.439^2^
	C				0.778^z^	0.011^3^

Abbreviations: C, controls; DM, diabetes mellitus; DMR, diabetic retinopathy; MMSE, Mini‐Mental State Exam; MoCA, Montreal Cognıtıve Assessment; pa, Kruskal–Wallis; pb, ANCOVA; pc, pTukey; p1, DM‐DMR; p2, DM‐C; p3, DMR‐C; px, group; py, education; pz, age; SD, standard deviation.

**TABLE 2:** Significant clusters showing altered gray matter volumes among the control, DM, and DMR groups.

	region of interest (ROI)	Region label in Destrieux cortical atlases	*p* value
**DMR < DM**	Right inferior temporal gyrus	rh_G_temporal_inf	.032
**DMR < Control**	Right insula (lateral and central part)	rh_G_Ins_lg_and_S_cent_ins_	.011

Abbreviations: DM, diabetes mellitus; DMR, diabetic retinopathy;

A significant negative correlation was observed between increased HbA1c levels and cognitive impairment involving the right insular region (*p* = 0.022). MoCA scores also exhibited significant correlation with the two regions, emerging as statistically significant in the analyses (right inferior temporal gyrus and right insula [lateral and central part]) (*p* = 0.003, *p* = 0.002) (Table 3). In contrast, no significant difference was observed in MMSE scores between the groups (Table 1).

**TABLE 3:** Correlations between morphometric alterations and clinical variables.

Partial correlation		rh_G_temporal_inf_ volume	rh_G_Ins_lg_and_S_cent_ ins_volume
HbA1c	Spearman's rho	−0.125	−0.416^*^
	*p* value	0.510	**0.022**
MoCA	Spearman's rho	0.498^**^	0.521^**^
	*p* value	**0.003**	**0.002**

Notes: Controlling for “eTIV”, “education”, and “age”.

* p < 0.05, ** p < 0.01, *** p < 0.001.

Abbreviations: eTIV, estimated total intracranial volume; MoCA, Montreal Cognıtıve Assessment.

## 5 Discussion

Although most studies have indicated the role of the hippocampus in impaired cognitive functions in T2DM, there are also rapidly replicating data showing a significant link between temporal lobe gray matter alterations and cognitive dysfunction (Moran et al. 2017). For example, Zhou et al. (2010) revealed a compensatory increase in temporalis inferior and middle regional connectivity in patients with T2DM resembling the compensatory network hyperactivity previously described in patients with mild cognitive impairment (MCI). This was confirmed by Chen et al. (2021), who showed critical alterations in temporal gray matter in patients with T2DM with cognitive impairment, representing a considerable risk factor for the development of dementia. The imaging data in the present research were consistent with our clinical results, particularly highlighting the role of right temporalis inferior and right insular regional volumes, which are associated with cognitive impairment in patients with T2DM (Xia et al. 2017).

A recent meta‐analysis indicated that the two regions described above were those most affected in DM‐related cognitive impairment (DM‐CI) patients, suggesting that the right hemispheric regions were more seriously impaired than the left sides in patients with DM. This represents a possible explanation of why right hemispheric‐related cognitive functions, such as visuospatial functions, are more seriously affected in patients with DM (Xia et al. 2017; Yao et al. 2021).

Later studies by Xiong et al. (2020) and Zhang et al. (2021) confirmed these results indicating right temporalis inferior involvement in cognitive impairment in patients with DM. Xiong et al. (2020) revealed marked impairment of network organization and ReHo values in the right temporalis inferior region in patients with DM with cognitive impairment compared to those without cognitive impairment. This interesting finding was confirmed in a further study showing that impaired inferior temporal regional functions were significantly correlated with MoCA scores in patients with DM. That research employed a novel imaging technique, voxel‐mirrored homotopic connectivity analysis. However, although there have been several studies implicating the right temporalis inferior region in DM‐CI, this has not been strongly confirmed for the right‐side insular region.

To the best of our knowledge, only a few studies have indicated that the right insular region is implicated in DM‐CI despite its well‐known role in cognition (Xia et al. 2017; Yao et al. 2021; Shao et al. 2022). For example, despite the lack of a significant association with cognition, Yang et al. (2016) found that the right insular region differed significantly between patients with DM‐CI and healthy controls. This was suggested by Shao et al.’s (2022), showing that patients with T2DM with CI exhibited significantly reduced bilateral gyrification and functional connectivity of the insular region correlated with general cognition and episodic memory scores. It is worth noting here that we have added value to the involvement of the right insular region in T2DM cognitive impairment, which Yang et al. (2016) discovered was impaired throughout the course of diabetes independently of cognition. Briefly, although these findings are generally consistent with the role of the temporalis inferior and insular region in cognition in healthy states, the literature regarding their specific association with cognition in diabetes is very scarce.

Our comprehensive analysis identified significant differences in the right temporal inferior and right insular regions between patients with DMR and the DM and control groups, a novel finding worthy of discussion. Particularly importantly, these regions exhibited significant differences and displayed significant correlations with MoCA scores. Notably, the right temporalis inferior region emerged as the only distinct region between the DM and DMR groups. Furthermore, the insular region was unique in correlating with both MoCA scores and HbA1c levels. To summarize, the fact that we were able to differentiate the DMR and control groups as well as the DM and the DMR groups based on clinical, imaging, and laboratory findings is an intriguing one. In particular, our observation of significantly lower cognition in the DMR group than in the DM group was unsurprising. That finding is consistent with previous research suggesting that DMR may represent a more complicated stage of cognition due to multiple end‐organ damage, particularly including the brain (Crosby‐Nwaobi et al. 2012).

In addition to suggesting that even raised HbA1c levels have a negative impact on cognition in healthy individuals, our findings are unique in demonstrating significant correlations among critical cognitive regions, glucose levels, and cognitive performances. From that perspective, it was unsurprising that our cohort was able to validate the well‐established connection between higher glucose levels and impaired cognition.

The finding that cognitive dysfunction and associated mild brain atrophy and symptomatic and asymptomatic infarcts has been already confirmed in non‐complicated type 2 diabetic individuals (Biessels and Despa 2018; Crosby‐Nwaobi et al. 2013; Li et al. 2018; Rodill et al. 2018), which places these patients at an elevated risk for overall cognitive dysfunction (Crane et al. 2013). This is supported by recent evidence linking not only DM but also prediabetes and modestly elevated glucose levels to the onset of dementia (Chen et al. 2015).

Although our findings suggest a progressive involvement of brain areas during the course of T2DM, we determined no significant difference in hippocampal volumes across the study groups, which may have been seen in the later phases of diabetes presenting with significant cognitive dysfunctions (Yao et al. 2021). Based on these findings, it may be postulated that the degeneration of key structures connected to core cognitive cortical regions occurs early during diabetes and before the development of retinopathy. In other words, the regions described above that also underwent substantial alterations in our study groups may comprise a crucial part of the cognitive network, fitting well with the critical regulatory role of the cortical circuits in specific cognitive functions (Zhou et al. 2010; Xia et al. 2017; Yao et al. 2021; Xiong et al. 2020).

Our observance of no significant differences in the occipital pole supports the idea that retinopathy has no effect on occipital lobe structural integrity, and that cognitive dysfunction is related to other critical structures apart from the indirect effect of retinopathy on cognition.

Despite the valuable cognitive and structural information yielded by the current study, the DMR group was relatively small, and a larger sample might have yielded more accurate results, particularly in terms of subtle structural and cognitive differences between the DM and DMR groups. However, a particular strength of this study is that we detected neuroimaging evidence for cognitive impairments, especially in the complicated diabetes phase, with abnormal levels of HbA1c. This suggests that early detection of the complicated phase of diabetes patients may be crucial for potential future interventions aimed at reducing these adverse effects, thus improving the management of these issues.

## 6 Conclusion

Our study suggests the marked presence of a neurodegenerative process associated with cognitive dysfunction in the patients with diabetic retinopathy. Further large‐scale studies are now needed to differentiate structural and cognitive parameters between diabetic patients with and without retinopathy.

## Author Contributions

**Ece Ozdemir Oktem**: conceptualization, resources, visualization, writing – original draft. **Dila Sayman**: investigation, writing – original draft. **Sevilay Ayyildiz**: formal analysis, software. **Çağlar Öktem**: data curation, resources, visualization. **Lutfiye Ipek**: formal analysis. **Behcet Ayyildiz**: software, formal analysis. **Fatih Aslan**: data curation. **Emin Utku Altindal**: data curation. **Nilay Yagci**: data curation. **Rumeysa Dikici**: formal analysis. **Ramazan Karaca**: investigation. **Şeyda Cankaya**: methodology, project administration, supervision, validation, writing – review and editing. **Seda Avnioglu**: data curation. **Halil Aziz Velioglu**: writing – review and editing, supervision. **Burak Yulug**: conceptualization, methodology, project administration, writing – original draft.

## Conflicts of Interest

The authors declare no conflicts of interest.

## Consent

Informed consent was received from all participants.

### Peer Review

The peer review history for this article is available at https://publons.com/publon/10.1002/brb3.70387

## Data Availability

The data that support the findings of this study are available on request from the corresponding author. The data are not publicly available due to privacy or ethical restrictions.
